# Immunogenicity of Plant-Produced Human Papillomavirus (HPV) Virus-Like Particles (VLPs)

**DOI:** 10.3390/vaccines8040740

**Published:** 2020-12-06

**Authors:** Paulina N. Naupu, Albertha R. van Zyl, Edward P. Rybicki, Inga I. Hitzeroth

**Affiliations:** 1 Biopharming Research Unit, Department of Molecular and Cell Biology, University of Cape Town, Rondebosch 7701, South Africa; naupupaulina@gmail.com (P.N.N.); ed.rybicki@uct.ac.za (E.P.R.); inga.hitzeroth@uct.ac.za (I.I.H.); 2 Institute of Infectious Disease and Molecular Medicine, University of Cape Town, Rondebosch 7701, South Africa

**Keywords:** cervical cancer, human papillomavirus, plants, immunogenicity

## Abstract

Cervical cancer is ranked fourth among the top cancers in women and is the second most common cancer in low- and middle-income regions, with ~570,000 new cases reported in 2018, which attributed to 84% of worldwide cervical cancer cases. Three commercially available prophylactic Human papillomavirus (HPV) vaccines are effective at preventing HPV infections. However, these vaccines are expensive due to their complex production systems, therefore limiting their use in developing countries. Recently, the use of plants to produce vaccines has emerged as a cost-effective alternative to conventionally used expression systems. Here, L1 proteins of eight high-risk (HPV 16, 18, 31, 33, 35, 45, 52, and 58) and two low risk (HPV 6 and 34) HPV types were successfully expressed in *Nicotiana benthamiana,* and transmission electron microscopy (TEM) analysis showed the presence of VLPs and/or capsomeres. Immunogenicity studies were conducted in mice utilizing HPV 35, 52, and 58 and showed that type-specific L1-specific antibodies were produced which were able to successfully neutralize homologous HPV pseudovirions in pseudovirion-based neutralization assays (PBNAs). This work demonstrated the potential for using plant-based transient expression systems to produce affordable and immunogenic HPV vaccines, particularly for developing countries.

## 1. Introduction

The HPV L1 capsid protein can self-assemble into virus-like particles (VLPs) that are structurally similar to native virions [1,2]. Interestingly, these VLPs can efficiently induce T-cell responses due to their particulate nature [3,4,5]. Furthermore, their surface has arrays of repetitive epitopes, which are recognized by B cells, making these VLPs highly immunogenic compared to other sub-unit vaccines [6].

Three prophylactic VLP-based HPV vaccines are commercially available: Cervarix™, Gardasil^®^, and Gardasil9^®^. Cervarix™ protects against HPV 16 and 18 infections, whereas Gardasil^®^ targets HPV 6, 11, 16, and 18 [7]. Gardasil9^®^ targets a wide spectrum of HPV types (HPV 6, 11, 16, 18, 31, 33, 45, 52, and 58) [8]. These vaccines are all formulated in aluminum-based adjuvants and studies have demonstrated that they are highly effective and safe.

HPV VLPs are produced in various systems, including bacteria, yeast, insect-cell, mammalian, and plant expression systems. For the production of the commercially available HPV L1 VLP-based vaccine Cervarix™, insect cells are used, while for the vaccines Gardasil^®^ and Gardasil9^®^ yeast is used. These vaccines are produced by subjecting purified L1 to in vitro disassembly/reassembly treatments, which results in VLP assembly and improves antigenicity, stability, and structural integrity of the VLPs [9]. These additional steps increase the already high expenditure of the overall production of VLPs in these systems [10].

Although the current commercially available HPV vaccines are effective at preventing HPV infections, the high costs associated with these vaccines prevents vaccination programs in developing countries, where they are most needed [11,12]. For example, Africa has the highest burden of cervical cancer [13], yet only 1–2% of girls aged 10–20 years get vaccinated in this region, with only 7 out of 54 countries having national HPV vaccination programs as of May 2018 [14,15]. In addition, some HPV types that have high prevalence in Africa, such as HPV 35, which is the fifth most prevalent type in Africa, are not included in the commercial vaccines.

Generally, the high costs of these vaccines are due to their complex production systems. Therefore, cost-effective alternative HPV vaccine production platforms need to be established for use in resource-poor countries. In recent years, plants have gained traction as cost-effective alternative production systems for recombinant proteins. Plant production platforms are safe, robust, and highly scalable, with low production costs and the eukaryotic machinery required for proper post translational modifications and assembly [16,17]. Therefore, using plant platforms to produce vaccines might overcome the disease burden in developing countries.

Plant expression systems can either be transgenic or transient. Recently, transient expression systems have gained attraction over stable transformed plants. This is because transient expression is in itself a contained system and can be used to rapidly produce large amounts of proteins, thereby saving time [17,18,19]. Studies that aimed to produce affordable HPV vaccines from plants mainly focused on the high-risk HPV 16 L1 and/or L1/L2 chimaeras [2,5,12,20,21,22,23,24,25,26] and low-risk HPV 11 L1 [27,28], which have been shown to elicit strong immune responses in animals.

Here, we investigated if plant-produced VLPs of important oncogenic HPV types could effectively induce a similar humoral immune response as Gardasil^®^. This was achieved by expressing the L1 proteins of eight high-risk HPV types (HPV 16, 18, 31, 33, 35, 45, 52, and 58) and two low risk types (HPV 6 and 34) in *N. benthamiana*. Furthermore, HPV 35, 52, and 58 were selected to assess the humoral immune response in mice. These were selected in particular, as HPV 35 is the fifth most prevalent type in Africa and HPV 52 and 58 are among the most frequently reported high-risk types in sub-Saharan Africa.

## 2. Materials and Methods

### 2.1. HPV Recombinant Plasmids

All the HPV L1 reference sequences of 6, 16, 18, 31, 33, 34, 35, 45, 52, and 58 were obtained from the PaVE website (http://pave.niaid.nih.gov) (GenBank accession number GI:60955, GI:333031, GI:60975, GI:333048, GI:333049, GI:9627334, GI:396997, GI:397022, GI:397038, and GI:222386, respectively). It was previously shown that a human codon optimized L1 gene resulted in higher accumulation of L1 when expressed in plants compared to expression of plant codon optimized and native L1 gene sequences [23]. Therefore, the L1 genes used in this study were human codon optimized and synthesized by GenScript.

The HPV L1 genes were obtained from GenScript in pUC57. All the gene sequences contained 5′ *Mlu*I and 3′ *Xho*l restriction enzyme sites that allowed for subcloning into the pTRAkc-rbcs1-CTP plant expression vector, a vector that targets expression to the chloroplast [23,29]. The L1 genes were excised from pUC57 using *Mlu*I/*Xho*I restriction enzymes and gel-purified (QIAquick^®^ gel extraction kit, Qiagen) after which they were directly ligated into pTRAkc-rbcs1-CTP, and linearized with the same restriction enzymes. The ligated constructs were transformed into competent *Escherichia coli* DH5α cells (Lucigen) and incubated overnight on Luria-Bertani (LB) plates supplemented with 100 µg/mL ampicillin at 37 °C. Recombinant clones were confirmed by colony PCR using vector specific primers: pTRA forward 5′-CATTTCATTTGGAGAGGACACG-3′ and pTRA reverse 5′-GAACTACTCACACATTATTCTGG-3′.

### 2.2. Transformation of Agrobacterium

The pTRAkc-rbcs1-CTP vector (as negative control) and pTRAkc-rbcs1-CTP-L1 plasmids were electroporated into electrocompetent *Agrobacterium* GV3101::pMP90RK cells according to the method described by Shen and Forde [30], plated onto LB agar plates supplemented with 30 μg/mL kanamycin, 50 μg/mL carbenicillin, and 50 μg/mL rifampicin, and incubated for 2–3 days. Positive clones were confirmed with colony PCR using the pTRA primers, back-transformation into *E. coli* DH5α and restriction enzyme digests.

### 2.3. Agrobacterium-Mediated Transient Expression in N. benthamiana

Expression of HPV 16 L1 using the pTRAkc-rbcs1-CTP plant expression vector had previously been optimized by our group [2,23,29], and these parameters were used for the expression of the L1 proteins in this study. Briefly, recombinant *Agrobacterium* constructs were inoculated into 10 mL LB medium supplemented with 30 μg/mL kanamycin, 50 μg/mL carbenicillin, and 50 μg/mL rifampicin and grown overnight at 27 °C with agitation. The following day, the 10 mL pre-inoculums were inoculated into bigger flasks, supplemented with the antibiotics mentioned above (except rifampicin) and 20 µM acetosyringone and incubated overnight as described above. Cultures were prepared for infiltration by diluting the overnight cultures to the required cell density of OD_600_ = 0.5 in resuspension buffer (5 mM MES, 10 mM MgCl_2_.6H_2_0, pH 5.6); 200 μM acetosyringone was added to the diluted culture. To allow induction of the *vir* genes by acetosyringone, diluted cultures were incubated at room temperature for an hour prior to infiltration. Leaves of 6-week-old *N. benthamiana* (~40 plants per HPV type) were vacuum infiltrated with the recombinant *Agrobacterium* cultures at –100 kPa vacuum pressure before releasing it. Before and after infiltration, plants were grown under the following conditions: 22 °C, 8 h dark, and 16 h of light. Biomass was harvested at 5 days post infiltration (dpi).

### 2.4. Extraction and Purification of VLPs

Whole leaves were homogenized with a T25 digital Ultra-Turrax^®^ (IKA^®^ Works Inc, Staufen, Germany) in 2x volumes of extraction buffer (1× High salt (0.5 M NaCl) Phosphate Buffered Saline, pH 7.4 [HSPBS] [23]), with cOmplete^TM^, ethylenediaminetetraacetic acid (EDTA)-free Protease Inhibitor, and incubated at 4 °C with agitation for 2 h. To remove plant debris, homogenates were filtered through four layers of 22–24 μm pore Miracloth™ (Millipore, Sigma, St. Louis, USA) and further clarified by centrifugation for 15 min at 15,317*× g*, 15 °C.

Clarified crude extracts were loaded onto 5 mL 30% (w/v) sucrose cushions overlaid onto 1 mL 50% sucrose cushions in 38 mL Ultra-Clear^TM^ ultracentrifuge tubes and centrifuged for 45 min at 174,587× *g*, 15 °C in a SW32Ti rotor (Beckman, Brea, CA, USA). All sucrose solutions were prepared in 1× HSPBS. After centrifugation, the 30% cushions were collected with a long needle and the sucrose removed by dialysing the samples using cellulose membrane dialysis tubing with 10 KDa cut-off (Sigma-Aldrich^®^, St. Louis, MO, USA) overnight at 4 °C in 1× HSPBS with agitation.

Dialyzed samples were loaded onto discontinuous Optiprep™ (Sigma-Aldrich^®^) density gradients (5 mL 27%, 4 mL 33%, 2 mL 39%, 1 mL 50%) prepared in 1x HSPBS and centrifuged for 3 h and 30 min at 174,587× *g*, 15 °C in a SW32Ti rotor (Beckman). After centrifugation, the bottom of the tubes were punctured, and 1 mL factions were collected.

### 2.5. SDS-PAGE and Western Blot Analysis

Purified L1 fractions were mixed with 1x sample application buffer (SAB; 0,001% (w/v) bromothymol blue, 0.5 M EDTA, 5% (w/v) SDS, and 25% (v/v) glycerol) and denatured at 95 °C for 5 min [31]. The L1 proteins were resolved on 10% SDS-PAGE gels at 120 V and either stained overnight at room temperature in Coomassie Blue stain (0.1% (w/v) brilliant blue G-250; 48% v/v methanol, 15% v/v glacial acetic acid) or subjected to western blot analysis. Coomassie-stained gels were destained with destain solution (30% v/v methanol, 10% v/v glacial acetic acid) overnight at room temperature. For western blot analysis, SDS-PAGE gels were transferred for 90 min at 15 V onto nitrocellulose membranes with a Trans-blot^®^ SD semi-dry transfer cell (Bio-Rad, Hercules, CA, USA). Membranes were blocked in blocking buffer (1× PBS; 5% long life fat-free milk, 1% Tween20) at room temperature for 30 min with agitation. The membranes were probed with 1:2000 rabbit-raised anti-Gardasil^®^ sera ^®^ diluted in blocking buffer by incubating at 4 °C overnight with agitation. The blots were washed 4 × 15 min in blocking buffer and incubated for 1 h at 37 °C with a 1:5000 dilution of anti-rabbit IgG alkaline phosphatase-conjugated secondary antibody (Sigma-Aldrich^®^) in blocking buffer with shaking. The membranes were washed 4 × 15 min with blocking buffer not containing milk and L1 proteins were detected with 5-bromo-4-chloro-3-indoxyl-phosphate (BCIP) and nitroblue tetrazolium (NBT) phosphatase substrate (BCIP/NBT 1-component, KPL) for 30 min.

### 2.6. Transmission Electron Microscopy (TEM)

TEM was used to determine if the expressed L1 proteins assembled into VLPs. A Model 900 SmartSet Cold Stage controller (Electron Microscopy Sciences, Hatfield, PA, USA) was used to glow discharge carbon-coated copper grids (mesh size 200) at 25 mA for 30 s. Samples were trapped onto grids by floating the grids, carbon side down, on the sample for 4 min. Thereafter, the grids were washed 4× with deionized water and negatively stained for 1 min with 2% w/v uranyl acetate. Grids were air-dried and viewed with an FEI Tecnai 20 transmission electron microscope.

### 2.7. Quantification of Vaccine Candidates

Three oncogenic HPV types (HPV 35, 52, and 58) were selected to immunize mice and were quantified and prepared for animal studies. These types were selected as they are relevant in an African context and are not included in or are cross-protected by Gardasil^®^.

Gel densitometry was used to quantify plant-produced HPV vaccine candidates; the concentration of L1 was determined relative to a Bovine serum albumin (BSA, [Sigma-Aldrich^®^]) protein standard. BSA standards and 25 µL of samples containing VLPs were mixed with 1× sample application buffer (SAB) and denatured at 95 °C for 5 min. Samples were loaded onto 10% SDS-PAGE gels and Coomassie-stained. Quantification of the protein bands were carried out with Studio^TM^ Lite version 5.2 software (LI-COR^®^, Lincoln, NE, USA).

After quantification, the negative control and L1 VLP samples were tested for endotoxin using a ToxinSensor™ chromogenic LAL endotoxin assay kit (GenScript, Piscataway, NJ, USA) according to the manufacturer’s instructions. Samples were also grown overnight on LB agar at 37 °C without antibiotics and observed for bacterial growth before being stored at −80 °C.

### 2.8. Immunization of Mice

The immunogenicity study was approved by the Animal Research Ethics Committee, Faculty of Health Sciences at the University of Cape Town (UCT, AEC 018-024). Fifteen 8-week-old female BALB/c mice were randomly divided into three groups of five each and housed in a biosafety level II (BSL-2) animal laboratory. Mice were acclimatized in the BSL-2 laboratory for 7 days prior to any experimental procedures. Mice were immunized with Gardasil^®^ (positive control), equimolar amounts of plant-produced HPV 35, 52, and 58 VLPs, or administered a purified empty vector as negative control; the concentration of the vaccines are summarized in Table 1. Gardasil^®^ has been previously shown to elicit a significant immune response in mice at 1/10th of a human dose [32]. In this study, 1/5th of a human dose was used. The plant-produced VLP vaccine candidates and empty vector negative control were emulsified in mineral oil MONTANIDE™ ISA 50 V2 adjuvant at a 60:40 (vaccine:adjuvant) ratio. MONTANIDE ISA 50 V2 is a ready-to-use oily vaccine adjuvant that has previously been used in mice [33,34].

### 2.9. Detection of Anti-L1 Antibodies in Mice Sera by Indirect Enzyme-Linked Immunosorbent Assay (ELISA)

#### 2.9.1. Pre-absorption of Mice Sera

Purified plant-produced HPV L1 proteins were used as coating antigens in ELISAs. It has been reported that host cell proteins (in this case co-purified plant proteins) present in the plant-produced vaccine candidates will elicit an antibody response in mice [2,5]. This was confirmed by initial ELISAs carried out on test and final bleeds, which showed high background detection of plant proteins in the empty vector negative control group serum.

To reduce the cross-reactivity of the antibodies present in the sera with host cell proteins the final bleeds from mice immunized with plant-produced L1 VLPs and the empty vector negative control were pre-absorbed using The “Lunchbox” Immunoabsorbent Technique [35], to potentially remove/reduce antibodies against native plant proteins present in the mice sera. Briefly, 6 g of uninfiltrated plant leaves were homogenized in 2× w/v HSPBS (pH 7.4) using a mortar and pestle. The crude extract was filtered through four layers of 22–24 μm pore Miracloth^TM^ (Millipore, Sigma, St. Louis, MO, USA) to remove plant debris. A piece of nitrocellulose membrane was incubated with the crude plant extract at 37 °C for 1 h with agitation. The membrane was washed 4× with blocking buffer (5% non-fat dairy milk (NFDM), 1× Tris-Cl [pH7.5]) before incubation overnight at 4 °C in 1:200 mice serum diluted in blocking buffer. The following day, the pre-absorbed sera were removed and used in all ELISAs in this study.

#### 2.9.2. Detection of Anti-L1 Antibodies in Mice Sera

For ELISAs, 96-well plates (Thermo Fisher Scientific, Waltham, MA, USA) were coated with 100 µL of the L1 antigens diluted to 0.8 ng/µl in coating buffer (10 mM Tris pH 8.5) and incubated overnight at 4 °C with agitation. Plates were blocked with 300 μL blocking buffer for 1 h at 37 °C, before they were washed 4× with 1× TST (0.05% Tween 20, 1× Tris-Cl pH 7.5). Diluted mice sera or rabbit-raised anti-Gardasil^®^ sera were added to the wells and the plates incubated for 1 h at 37 °C. The plates were washed as described above and 100 µL of 1:5000 dilution of alkaline phosphatase-conjugated anti-mouse IgG secondary antibody and anti-rabbit IgG for Gardasil^®^ (Sigma-Aldrich^®^) were added to each well and the plates incubated for 1 h at 37 °C. Plates were washed 4× with (1× Tris-Cl, pH 9.0), after which 200 μL SIGMAFAST™ *p*-nitrophenyl phosphate (Sigma-Aldrich^®^) substrate was added to each well. The plates were incubated in the dark for 30 min before reading the absorbance with a Bio-Tek Powerwave XS spectrophotometer at 405 nm.

Controls included: wells coated with coating buffer (to obtain background readings), pre-bleeds (negative controls), and rabbit-raised anti-Gardasil^®^ sera (positive control). A plant-produced empty vector was used as negative control for plant-made vaccine candidates only. ELISA data were all normalized by subtracting the background readings and titres are stated as the reciprocal of the maximum dilution with higher absorbance readings than the equivalent pre-bleed serum. Moreover, serum obtained from mice vaccinated with the plant-produced VLPs that produced titres with absorbance readings higher than the empty vector at the lowest dilution (1:200) were considered anti-L1 positive.

### 2.10. Pseudovirion-Based Neutralization Assays

HPV pseudovirion (PsV) production, purification, and neutralization were all done as described by Buck and co-workers [36], with a few adjustments as described by Pineo and co-workers [5]. PsVs were made in HEK293TT cells for HPV 6, 16, 18, 31, 35, 45, 52, and 58. Sera that resulted in 50% reduction of secreted embryonic alkaline phosphatase (SEAP) signals when compared to a PsV-only control were considered neutralizing positive and were further titrated to determine the endpoint of neutralizing titres. All raw data were normalized for background by subtracting the cells only data; thereafter, PsV-only data were set as the maximum (100%) SEAP signal. For each HPV type, neutralization is expressed relative to its corresponding PsV-only control (no neutralization), and data plotted as the percentage of the relative light unit (% RLU).

## 3. Results

### 3.1. Expression and Purification of HPV L1 VLPs

**Figure 1:**
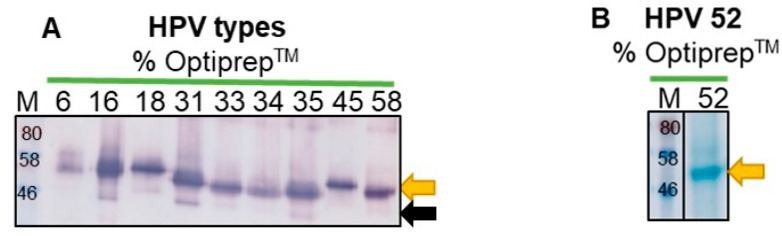

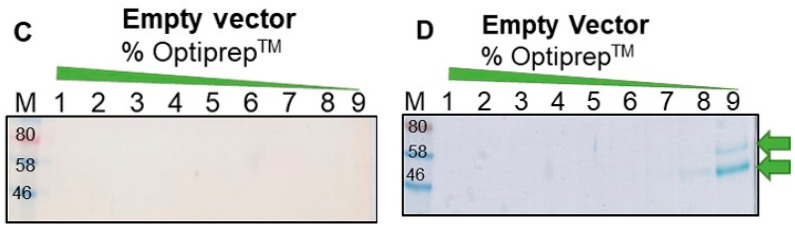
Analysis of plant produced HPV VLPs: (**A**) western blot of all HPV types detected with anti-Gardasil^®^; (**B**) Coomassie-stained gel for HPV 52; (**C,D**) western blot and Coomassie-stained gel for the empty vector, respectively. Purified HPV L1 proteins (~56 kDa) were probed with rabbit-raised anti-Gardasil^®^ sera (1:2000 dilution) and anti-rabbit IgG alkaline phosphatase-conjugated secondary antibody (1:5000). Labels: M: pre-stained protein standard (kDa); yellow arrows: HPV L1 protein (~56 kDa); green arrows: native plant proteins; black arrow: HPV L1 cleavage product.

In this study, large scale expression of the L1 proteins were carried out by vacuum-infiltrating *N. benthamiana* plants with recombinant *Agrobacterium* cultures at a final OD_600_ of 0.5. Plants were harvested at 5 dpi, biomass presented with slight chlorosis in plants infiltrated with the HPV L1 constructs compared with the negative control, pTRA-rbcs1-cTP-only plants. In all cases, no tissue necrosis was observed. L1 expression was confirmed on western blots and a Coomassie-stained gel, in the case of HPV 52 L1, after Optiprep™ density-gradient purification. The L1 proteins of the 10 HPV types were successfully expressed in *N. benthamiana* and purified. This is evidenced by the detection of L1 bands (~56 kDa) for all HPV types (yellow arrows, Figure 1A,B). For HPV 16, 18, 31, and 35, a second band was detected at ~46 kDa, indicating potential cleavage products of L1 (Figure 1A, black arrow). HPV 52 was analyzed on Coomassie-stained SDS-PAGE only, because there was no antibody to reliably detect HPV 52 L1 on western blot. The Coomassie-stained gel indicated the presence of HPV 52 L1 at ~56 kDa (Figure 1B).

The empty vector (pTRAkc-rbcs1-CTP) negative control was analyzed on a western blot and Coomassie-stained gel and, as expected, no L1 bands were observed on the western blot (Figure 1C,D). Two bands, at ~59 and 55 kDa, were observed in fraction 9 on the Coomassie-stained gel of the negative control sample, possibly indicating the presence of native plant proteins, such as ribulose-1,5-bisphosphate carboxylase/oxygenase (RuBisCo), in less dense gradient fractions (Figure 1D).

**Figure 2:**
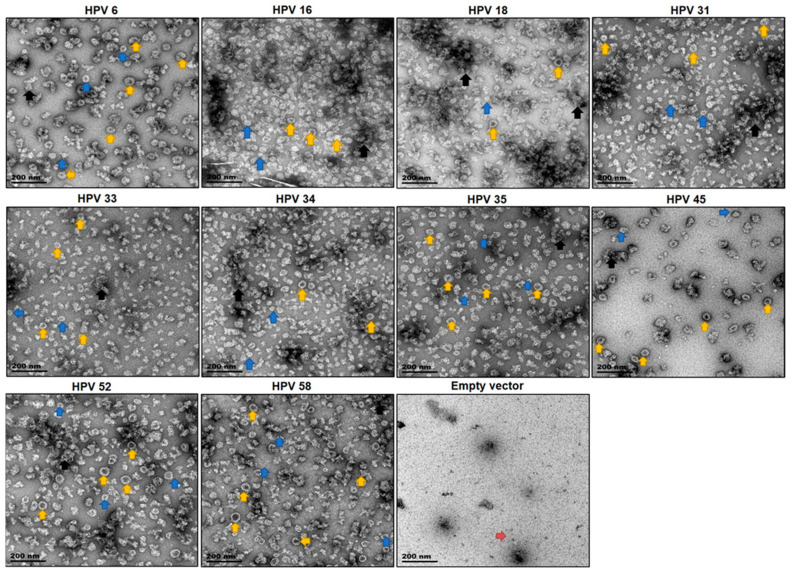
Transmission electron micrographs of purified VLPs. VLPs were purified on Optiprep™ density gradients and viewed under EM at a 40,000 × magnification. Scale bars are 200 nm in size. Arrows point to representative examples of VLPs (yellow arrows), small VLPs and/or capsomeres (blue arrows), L1 aggregates (black arrows), and native plant aggregates (red arrow).

TEM analysis of purified fractions of each HPV type showed fully assembled spherical VLPs measuring 40–60 nm diameter (Figure 2, yellow arrows), small VLPs and/or capsomeres of 25–39 nm diameter (Figure 2, blue arrows) and potential L1 aggregates (Figure 2, black arrows). No structures resembling VLPs and/or capsomeres were observed in the purified negative control samples, structures seen in the negative control are probably plant aggregates (Figure 2, red arrow). VLPs were comparable to HPV VLPs that had previously been produced in our group [2,5,29]. By comparing VLPs per field of view, the expression of HPV 6, 16, 35, 45, 52, and 58 L1 resulted in the assembly of more fully formed VLPs (40–60nm) as compared to HPV 18, 31, 33, and 34, which were mostly populated by capsomeres and aggregates. Moreover, more uniform VLPs were observed for HPV 52 and 58, with an average of more than 80 VLPs counted per field of view compared to an average of 40–78 VLPs per field of view for HPV 6, 16, 18, 31, 33, 34, and 35. HPV 45 had the lowest number of VLPs with an average of 14 VLPs counted per field of view.

### 3.2. Determination of Anti-L1 Titres

#### 3.2.1. ELISA Analysis

**Figure 3:**
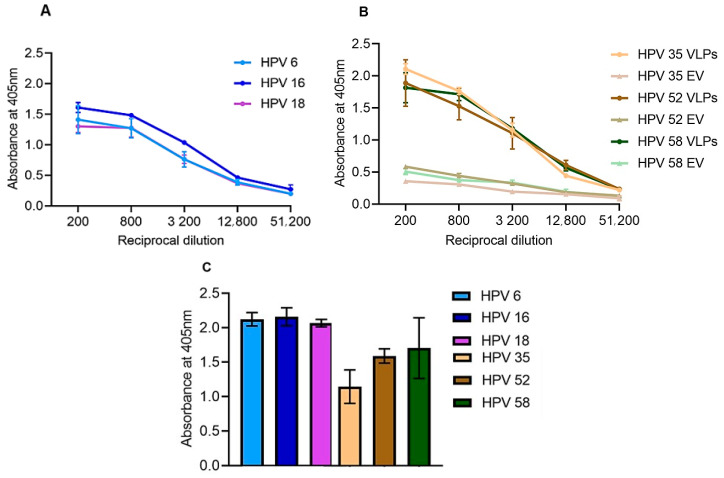
Indirect ELISA to determine anti-L1 titres. Plates were coated with purified plant-produced L1 antigens and final bleed antiserum collected from mice vaccinated with Gardasil^®^, plant-produced VLPs and the empty vector negative control (EV) were titrated to determine end-point anti-L1 titres. (**A**) Four-fold serial dilution of pooled sera from mice vaccinated with Gardasil^®^ and (**B**) plant-produced EV and VLP respectively; (**C**) Plant-produced HPV VLPs were probed with rabbit-raised anti-Gardasil^®^ positive control sera at a single dilution of 1:5000 to determine if the plant-produced VLPs could be detected by Gardasil^®^ antisera. Markers and error bars indicate mean values and standard deviation from triplicate readings.

To determine whether plant-produced HPV VLPs can elicit a humoral immune response in mice as efficiently as the commercially available Gardasil^®^, mice were immunized with a total of 6 µg of plant-produced VLPs or 1/5th of the human dose of Gardasil^®^ (total amount of 24 µg). The humoral immune response was analyzed by testing type-specific anti-L1 antibodies in ELISA using plant-produced VLPs as antigen. Sera from individual mice in each group were pooled and anti-L1 antibody titres determined by indirect ELISAs (Figure 3). The commercial Gardasil^®^ vaccine elicited a significant immune response, with the highest anti-L1 titre of 12,800 observed for HPV 6, 16, and 18 (Figure 3A). Plant-produced L1 VLPs also elicited a significant humoral immune response against type-specific HPVs (Figure 3B). However, titres of 200–3200 were observed in sera of mice vaccinated with the empty vector control, which may have been due to antibodies against contaminating plant proteins in the vaccine preparations (Figure 3B). Therefore, a cut-off absorbance of OD 0.5 was determined for plant-produced VLPs and resulted in the highest anti-L1 titres of 12,800 for all three HPV types (Figure 3B). No anti-L1 response was observed in the pre-bleed serum of all the groups. As a positive control in the ELISA, wells coated with plant-produced VLPs were detected with rabbit-raised anti-Gardasil^®^ antibody (1:5000), which confirmed the presence of antigens in the coated samples and validated the experiments (Figure 3C).

#### 3.2.2. Detection of Anti-L1 Neutralizing Antibodies in Mice Sera

**Figure 4:**
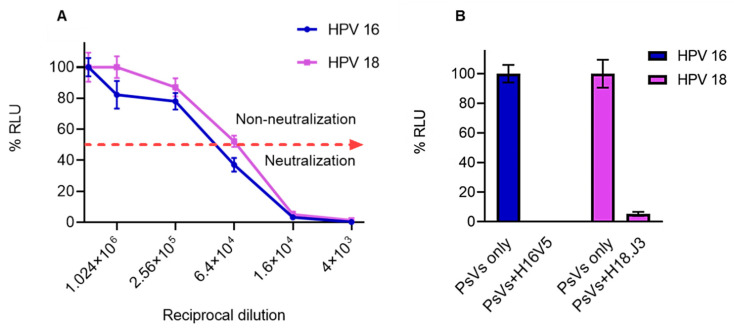

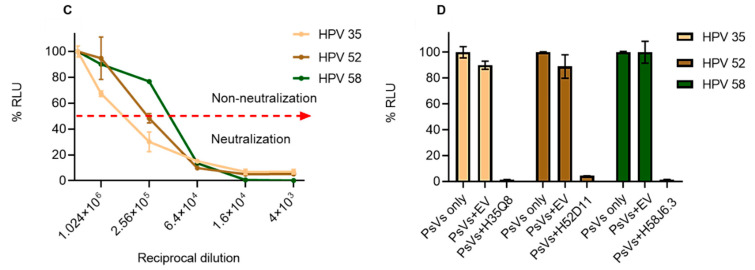
In vitro PsV neutralization titres in L1 PBNA. Purified PsVs of HPV types 16, 18, 35, 52, and 58 were used in L1-PBNAs to detect L1-specific neutralizing antibodies in sera obtained from vaccinated mice. (**A**) Neutralization of HPV 16 and 18 PsVs with pooled Gardasil^®^ mouse sera, the cut off for anti-L1 neutralizing titres was a 50% reduction of SEAP signal (dashed red line). (**B**) Positive control monoclonal antibodies H16V5 and H18.J3 were able to neutralize HPV 16 and 18 PsVs completely. (**C**) Pooled sera from mice vaccinated with plant-produced VLPs tested for neutralization of homogenous PsV types (HPV 35, 52 and 58). The red dashed line indicates the cut off for anti-L1 neutralizing titres (50% reduction of SEAP signal). (**D**) Positive and negative control neutralization of PsVs: empty vector (EV) negative, positive control monoclonal antibodies (H35Q8 for HPV 35, H52D11 for HPV 52, and H58J6.3 for HPV 58). Markers and error bars indicate mean values and standard deviation from triplicate readings.

L1 PBNAs were carried out to test the ability of sera obtained from mice to neutralize HPV PsVs. Well-characterized neutralizing antibodies (H16V5, H18.J3, H35Q8, H52D11, and H58J6.3), provided by Neil Christensen [37], were used as positive controls in the PBNAs. The Gardasil^®^ vaccine has been studied extensively and shown to elicit homologous and heterologous neutralizing antibodies [38,39]. Therefore, the neutralization ability of pooled Gardasil^®^ mouse sera was only tested on HPV 16 and 18 PsVs in this study. HEK293TT cells transfected with PsVs carrying a SEAP plasmid reporter gene were preincubated with antibodies. The pooled sera successfully neutralized both HPV 16 and HPV 18 PsVs, the cut off for anti-L1 neutralizing titres was a 50% reduction of SEAP signal (Figure 4A, dashed red line). Serum neutralized HPV 16 and 18 PsVs at neutralization titres of 4000–16,000 by ~97%. The highest neutralization titre observed was 64000, which neutralized HPV 16 PsVs (˂50% SEAP signal observed), but not HPV 18 PsVs (>50% SEAP signal detected) (Figure 4A). This suggested that the serum had more potent neutralizing antibodies for HPV 16 compared to HPV 18. This was not surprising, as the vaccine contained a higher concentration of HPV 16 L1 (8 µg) than HPV 18 L1 (4 µg). However, no statistically significant difference in the neutralization ability between HPV 16 PsVs and HPV 18 PsVs, *p* > 0.05 (*p* = 0.3191), was observed.

Sera from mice immunized with plant-produced VLPs successfully neutralized type-specific PsVs (HPV 35, 52 and 58) (Figure 4C). Neutralization of ~95% was observed at titres of 1000–16,000 for all the type-specific PsVs. Furthermore, ~91% neutralization was observed at titres of 64000 for all HPV PsVs types (Figure 4C). The highest neutralization titre of 256,000 was observed for HPV 35 and 52 PsVs, 50% cut off (red-dashed line, Figure 4C). However, there was no statistically significant difference in the neutralization ability between these types (*p* = 0.1436).

Negative controls for the PBNAs included PsVs that were not treated with sera and PsVs treated with sera from mice vaccinated with the empty vector, and no neutralization was observed in the empty vector sera. (Figure 4B,D). Treatment of PsVs with type-specific positive control monoclonal antibodies (H16V5 for HPV 16, H18.J3 for HPV 18, H35Q8 for HPV 35, H52D11 for HPV 52, and H58J6.3 for HPV 58) resulted in 100% neutralization when compared to the negative control PsVs only (Figure 4B,D). The positive neutralizing antibody controls successfully neutralized their corresponding PsVs, showing the validity of the assays.

To our knowledge, there is no evidence of cross protection of heterologous HPVs by HPV 35, 52, or 58. However, we wanted to investigate if sera obtained from mice vaccinated with plant-produced VLPs were able to cross protect against other HPV types, and therefore, sera were tested in PBNAs with heterologous PsVs that were available in our lab (HPV 6, 16, 18, 31, and 45), to determine if they could neutralize these PsVs. Unfortunately, plant-produced serum did not neutralize any of heterologous PsVs tested in this study at a 1:50 dilution of the sera. Highest neutralization titres for all sera are summarized in Table 2.

## 4. Discussion

HPV 16 L1 and/or L1/L2 chimaeric VLPs have previously been expressed in plants and have been shown to elicit immune responses in animal models [2,5,22,23,24]. Most recently VLPs for high and low-risk HPV types were successfully produced in plants [29]; however, immunogenicity of these VLPs were not assessed. In this study, 10 HPV types were transiently expressed in *N. benthamiana* and the immunogenicity of three of these, which were important in an African context, was analyzed in mice.

Ten HPV types were successfully expressed in *N. benthamiana* using the pTRAkc-rbcs1-CTP plant expression vector that targets expression to the chloroplast [23]. Expression of L1 was confirmed on western blots for HPV 6, 16, 18, 31, 33, 34, 35, 45, and 58 (and a Coomassie-stained gel for HPV 52) with the detection of bands at ~56 kDa (Figure 1, yellow arrow). These results are consistent with other studies [2,5,12,22,23,29]. For HPV 16, 18, 31, and 35, bands were detected at ~46 kDa on western blots; these could potentially be proteolytic cleavage products of L1 (bands ~46 kDa) (Figure 1, black arrow). Protein degradation in tobacco plants have been reported in other studies [2,22,40,41,42], which may be explained by an abundance of proteases that are present in plants. Proteins can be degraded either inside the cells by intracellular proteases or outside by extracellular proteases [41,43]. Among other proteases, the genome of tobacco is known to code for a minimum of 60 putative cysteine proteinases (CysPs), which might be involved in protein degradation [44,45].

Despite potential proteolytic cleavage occurring for some of the L1 proteins expressed in this study, TEM analysis showed the presence of fully assembled VLPs measuring 40–60 nm in diameter as well as small VLPs and/or capsomeres measuring 25–39 nm (Figure 2). HPV L1 has been shown to assemble into particles ranging from 25–60 nm in different expression systems, including plants [2,5,12,23,27,46]. Full sized (40–60 nm) and small (25–39 nm) plant-produced HPV 16 L1 VLPs have previously been observed in the chloroplast [2,5,12,23]. The presence of L1 aggregates after purification was not surprising, as it appears that recombinantly produced HPV L1 seems to be prone to aberrant assembly, since the presence of aggregates has been reported in other research [2,12,23,27]. However, the formation of L1 aggregates may not be unique to plants; HPV VLPs produced in other expression systems—such as yeast and insect cells—are made by in vitro disassembly/reassembly of purified L1 to obtain a homogenous population of VLPs, suggesting the formation of aggregates by these systems [47,48].

HPV VLPs are highly immunogenic because of their repetitive surface-displayed epitopes, ability to activate B-cells, their particulate nature, and their size, which is ideal for the uptake of nanoparticles by dendritic cells [49,50]. In this study, mice were vaccinated with plant-produced VLPs for HPV 35, 52, and 58 to determine if neutralizing antibodies were elicited by the plant-produced VLPs.

ELISA (Figure 3) analysis showed that anti-L1 immune responses were successfully elicited in mice vaccinated with the commercial Gardasil^®^ vaccine and the plant-produced trivalent VLP vaccine candidate, whereas no L1 response was detected in the negative control empty vector sera. PBNA was used to investigate the ability of the VLP vaccines to neutralize PsVs. Sera from mice immunized with the trivalent VLP vaccine candidate elicited type-specific neutralizing antibodies, which successfully neutralized HPV 35, 52, and 58 PsVs (Figure 4). Neutralizing antibody titres of 4000–64,000 for HPV 58 and 4000–256,000 for HPV 35 and 52 were observed (Figure 4C). These titres were ~40-fold higher than titres reported for plant-produced HPV 16 against type-specific PsVs in other studies [2,5,22,23]. This being said, mice in this study were hyper-immunized and received five vaccinations, which may explain the high titres observed here. The ability of sera to prevent cell infection by PsVs have been associated with defense against experimental and natural infections [51]. Therefore, plant-derived HPV VLPs have the potential to be used as a prophylactic vaccine, which might alleviate cervical cancer and related diseases in resource-poor countries.

No neutralization was observed against heterologous HPV 6, 16, 18, 31, and 45 PsVs in this study. However, this was not surprising, as there is no evidence of cross-protection between these HPV types. HPV types are grouped according to their phylogenetic similarity, based on the L1 capsid sequences. Because they are phylogenetically related, different HPV types can share epitopes on the capsid, which can elicit a cross-reactive immune response [52,53,54]. The HPV types expressed in this study belong to the genus *Alphapapillomavirus*, which could explain why the rabbit-raised Gardasil^®^ antiserum was able to detect all the plant-produced HPV types on western blots, except for HPV 52 (Figure 1), which could only be detected by ELISA (Figure 3C). This is potentially because antibodies in the Gardasil^®^ antiserum cross-reacted with conformational epitopes on HPV 52 VLPs that were destroyed upon denaturing of the protein for western blot analysis. The data presented here highlight that detection of protein on western blots and ELISAs, does not necessarily infer functionality in terms of neutralization or cross-neutralization of viral particles. This is why sera from mice vaccinated with plant-produced HPV 35, 52, and 58 VLPs did not neutralize heterologous PsVs tested in the PBNAs.

The immune response elicited by the plant-produced trivalent VLP vaccine candidate cannot be directly compared to that of Gardasil^®^ for different reasons. Firstly, these vaccines were produced in different expression systems, by different purification methods, and were formulated with different adjuvants, all of which could have played a role in the immunogenicity of the vaccines. Secondly, different doses were used for the two vaccine candidates. Gardasil^®^ was administered at 1/5th of the human dose (24 µg total L1), while plant-produced trivalent vaccine was used at 6 µg (total L1). However, the plant-produced trivalent vaccine candidate induced a humoral immune response comparable to the immune response elicited by the commercially available HPV Gardasil^®^ vaccine. This was evidenced by the type-specific anti-L1 neutralizing titres observed (Figure 4), suggesting that plant-produced VLPs are suitable for making HPV vaccines.

## 5. Conclusions

In conclusion, we demonstrated the successful production of a trivalent plant-produced VLP vaccine candidate consisting of HPV 35, 52, and 58. The vaccine successfully elicited a type-specific humoral immune response with neutralizing antibodies. This work has successfully demonstrated that plant-produced HPV VLPs are highly and appropriately immunogenic, demonstrating the potential to use plant-based transient expression systems to produce cost-effective HPV VLP-based vaccines, particularly for developing countries.

## Figures and Tables

**Table 1:** Vaccine candidates used to immunize mice in this study.

Vaccine Group	Vaccine Name	HPV L1 Content	Adjuvant	# of Mice	L1 Dose µg/100 µL	Total L1 Dose µg/100 µL
Group 1	Gardasil^®^	6, 11, 16, and 18	Amorphous aluminum hydroxyphosphate sulfate	5	HPV 6–4 µg HPV 11–8 µg HPV 16–8 µg HPV 18–4 µg	24 µg (1/5th human dose)
Group 2	Plant-made VLPs	35, 52, and 58	MONTANIDE™ ISA 50 V2	5	HPV 35–2 µg HPV 52–2 µg HPV58–2 µg	6 µg
Group 3	Purified empty vector	-	MONTANIDE™ ISA 50 V2	5	-	-

Three days prior to the first immunization (day 0), pre-bleeds were collected from individual mice. Mice were subcutaneously injected with 50 mL of the vaccine candidates into both the left and the right flank on day 3, 17, 31, and 45 (every 2 weeks), before obtaining the test bleeds on day 56. An additional boost inoculation was administered on day 59, before obtaining the final bleeds on day 73.

**Table 2:** Summary of in vitro PsV neutralization titers in L1 PBNA.

Neutralization Titre					
	HPV 16	HPV 18	HPV 35	HPV 52	HPV 58
Gardasil^®^ sera	≤64,000	<64,000	-	-	-
H16V5	5000	-	-	-	-
H18.J3	-	10,000	-	-	-
Plant-produced VLPs sera	-	-	≤256,000	≤256,000	<256,000
H35Q8	-	-	500	-	-
H52D11	-	-	-	100	-
H58J6.3	-	-	-	-	20 000
Empty Vector	NN	NN	NN	NN	NN
PsVs only	NN	NN	NN	NN	NN

Assays were performed in triplicate. NN: No neutralization, -: Not applicable.
